# Special Issue on ‘Coronavirus: Vaccines and Other Therapeutics’ (2020–2021)

**DOI:** 10.3390/vaccines9101083

**Published:** 2021-09-26

**Authors:** Sankar Basu

**Affiliations:** 1 Department of Microbiology, Asutosh College, 92, Shyama Prasad Mukherjee Rd, Bhowanipore, Affiliated to Calcutta University, Kolkata 700026, India; nemo8130@gmail.com; 2 3BIO Group (External Scientific Collaborator), ULB, 1050 Brussels, Belgium

As is well known, the emergence of SARS-CoV-2 ever since late 2019 [1] and its growing list of variants [2,3,4] are still an alarming concern to global health and economy. Painstaking efforts are being devoted worldwide against all odds to find a sustainable solution spending huge amount of money and relentless labor. The efforts have already resulted in the development of several approved vaccines [5,6,7] which are being recommended and administered at a mass-scale by governments in and across countries and continents. In parallel, several plausible alternative therapeutics have also been proposed, mostly based on rationalized computational predictions [8,9,10], some of which have even passed their wet-lab validation tests and early rounds of clinical trials [11] in global academia. The re-purposing of known drugs [12,13] and/or earlier vaccines have also been attempted and so has been antisera from convalescent patients in order to procure templates of efficient antibodies for further modulation and structural optimization [14,15]. In contrast to these success stories, however, the pandemic and its therapeutic endeavors have also raised controversies and debates all around the globe, starting from the very origin of the virus (whether natural or human-intervened) to the ethical grounds of the ongoing vaccination policies.

Especially regarding the ‘true’ origin of SARS-CoV-2, alternative theories have surfaced up with impressive counter-narratives and a growing body of eye-opening rationales (supported by latest experimental findings) to that of the fairly well believed ‘natural origin theory’ [16,17] accepted in academia in the early days of the pandemic. Independent re-investigations of the viral origin are, thus, of high demand at the moment and also with the review of policies in ‘gain of function’ virology research. The latest understanding is that it was during such a systematic ‘gain of function’ mutational studies [18] carried out on gradually evolving strains of the coronavirus (starting from its natural template, SARS-CoV, 2003) that the virus triggering the current pandemic (SARS-CoV-2) accidentally got released from a renowned biosafety-level-4 virology laboratory at the Wuhan Institute of Virology, Wuhan, China (the ‘lab-escape’ theory). Genome comparison studies of the related lineage of respiratory viruses of late indicate that SARS-CoV-2 is likely to be chimeric and further reveals the presence of a unique Furin-like cleavage site (FLCS) in its Spike (FLCS_Spike_, absent in other related respiratory viruses), which has undoubtedly shaken the scientific community for a plethora of reasons. Even satirical phrases such as the ‘smoking gun’ have been coined [19] referring to the arginine-reach ‘PRRAR’ motif at the heart of this FLCS_Spike_ indicating plausible devastating consequences in the massively elevated transmissibility in COVID-19 compared to earlier onsets of respiratory viral diseases. The emergence of this FLCS_Spike_ can also been envisaged as a major shift in focus from a (bio-)physics-(the non-covalent RBD_Spike_–hACE2 interaction) to a (bio-)chemistry-(the Furin-cleavage of Spike involving breaking of covalent chemical bond(s)) window in COVID-19 research [19]. From a mechanistic end, the FLCS_Spike_ also seem to be unique in harboring a characteristic loop-disorder, intrinsic to the high positive charge cloud heavily localized at the ‘PRRAR’ motif and also to undergo a ‘disorder-to-order transition’ upon binding to Furin, enabling efficient proteolytic cleavage at the Spike-S1/S2 interface. Coordinates of the concerned FLCS_Spike_ patch being missing in all experimental structures of the SARS-CoV-2 Spike [20,21,22] strongly speaks in favor of the loop-disorder while the ‘targeted’ cleavage by Furin at the FLCS_Spike_ appears to be the causal factor in the drastic increase in viral transmissibility. All said, the origin of the virus still remains obscure and debatable.

From a therapeutic point of view, diverse groups of people worldwide have had to deal with the question of authenticity regarding information floating over social and electronic media in the past couple of years. This has certainly indulged doubts in the mind of many people regarding (for example) vaccination, whether safe or not, whether effective or not and so on. Even with this handicap, a wide variety of main-stream therapeutics (referring primarily to the approved vaccines) have been procured, recommended and administered globally, resulting in 30.5% of the global human population of 7.9 billion being fully vaccinated while 42.8% being partly (dated 15 September 2021 [23]). Alongside with these mainstream approaches, alternative therapeutic directions (including ‘reverse’ approaches [9]) have also being explored (Table 1), presenting a whole spectrum from the design of mini-proteins [8], peptide blockers [24], nanobodies [10], RBD_Spike_ structural mimics [9], influenza virus-like particle (VLP) vaccines [25] and possibly others. Studies on natural products [26,27,28] promised to serve as possible ‘herbal medicines’ for the future have also been reported in great number.

This Special Issue themed on “Coronavirus Vaccines and Other Therapeutics” is focused on high-quality research in any basic and/or applied area related to the ongoing pandemic. We welcomed articles of various formats, ranging from high-quality reviews, regular research papers and communications to brief reports contributing either fundamentally or clinically to COVID-research. In addition to the standard vaccination approach, we categorically encouraged studies on alternative therapeutic endeavors, e.g., other modes of immunization, antigen arrest and novel inhibitors. We were also considerably open to debatable topics, both related to the viral origin and on various matters of (non-standard) therapeutic approaches. This, however, does not mean any major shift in the long-term agenda of the journal, which is primarily centered on laboratory and clinical vaccine research, utilization and immunization. We sincerely hope that, in this time of crisis, the Special Issue favorably contributes to academia and, more so, to the translational research of scientific laboratories across the globe.

## Figures and Tables

**Table 1:** Look-up table portraying the range of therapeutic approaches to combat the ongoing Coronavirus pandemic.

Covid-19 Therapeutic Approaches		
Main-Stream Approaches	Alternative Approaches	
Vaccines (whole-organism/subunit vaccines, semi-synthetic/recombinant vaccines, m-RNA/protein-based, thermotolerant)	Designed Molecules	Natural Products
	Mini-Protein	Plausible future-herbal medicines
	Peptide blockers	
	Nanobodies	
	RBD_Spike_ structural mimics (non-virulent)	
	VLP vaccines	
	Others	

## Data Availability

Not applicable.
